# Caffeine Encapsulation in Metal Organic Framework MIL-53(Al) at Pilot Plant Scale for Preparation of Polyamide Textile Fibers with Cosmetic Properties

**DOI:** 10.1021/acsami.2c04293

**Published:** 2022-05-04

**Authors:** Beatriz Zornoza, César Rubio, Elena Piera, Miguel A. Caballero, Daniel Julve, Jorge Pérez, Carlos Téllez, Joaquín Coronas

**Affiliations:** † Instituto de Nanociencia y Materiales de Aragón (INMA), Universidad de Zaragoza-CSIC, 50009, Zaragoza, Spain; ‡ Chemical and Environmental Engineering Department, Universidad de Zaragoza, 50018, Zaragoza, Spain; § Research and Development Department. Nurel S.A., Ctra. Barcelona km 329, 50016, Zaragoza, Spain; ∥ Industrias Químicas del Ebro (IQE) S. A. Grupo IQE, 50016, Zaragoza, Spain

**Keywords:** Metal organic framework, MIL-53(Al), carboxylate ligand, scaled-up synthesis, caffeine, microencapsulation, textile fiber, polyamide

## Abstract

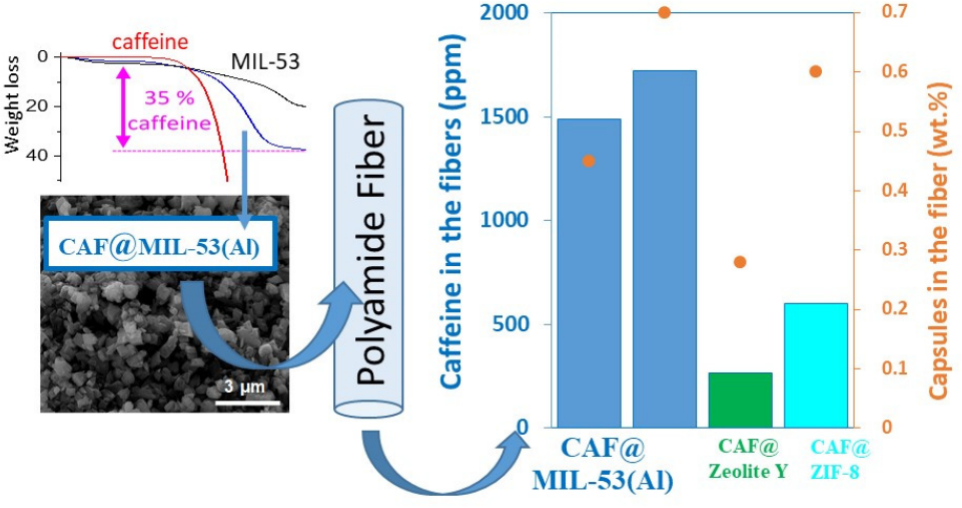

Currently in the marketplace, we can find clothing items able to release skin-friendly ingredients while wearing them. These innovative products with high-added value are based on microencapsulation technology. In this work, due to its lightness, flexibility, porosity, chemical affinity and adsorption capacity, metal–organic framework (MOF) MIL-53(Al) was the selected microcapsule to be synthesized at a large scale and subsequent caffeine encapsulation. The synthesis conditions (molar ratio of reactants, solvents used, reaction time, temperature, pressure reached in the reactor and activation treatment to enhance the encapsulation capacity) were optimized by screening various scaling-up reactor volumes (from lab-scale of 40 mL to pilot plant production of 3.75 L). Two types of Al salts (Al(NO_3_)_3_·9H_2_O from the original recipe and Al_2_(SO_4_)_3_ as commercial SUFAL 8.2) were employed. The liporeductor cosmetic caffeine was selected as the active molecule for encapsulation. Caffeine (38 wt %) was incorporated in CAF@MIL-53(Al) microcapsules, as analyzed by TGA and corroborated by GC/MS and UV–vis after additive extraction. CAF@MIL-53(Al) microcapsules showed a controlled release of caffeine during 6 days at 25 °C (up to 22% of the initial caffeine). These capsules were incorporated through an industrial spinning process (with temperatures up to 260 °C) to manufacture PA-6 fibers with cosmetic properties. Up to 0.7 wt % of capsules were successfully incorporated into the fibers hosting 1700 ppm of caffeine. Fabrics were submitted to scouring, staining, and washing processes, detecting the presence of caffeine in the cosmetic fiber.

## 1 Introduction

Microencapsulation technology is a growth area in the textile industry.^1^ In addition to behaving like conventional fibers, fibers containing microcapsules also show different features regarding the encapsulated specific additives incorporated in the capsules, highlighting the innovation and development of such value-added materials.^2^ A broad variety of ingredients, including antimicrobials, phase change materials, antibiotics, vitamins, fragrances, insect repellents, dyes, or flame retardants, are interesting for textile manufacturers to be encapsulated.^1^ Among them, caffeine is an amphiphilic additive with significant lipolytic capacity, used as a fat reducer, with its main field of application being the cosmetic and pharmacology industries.^3^

Even though the microencapsulation process is commonly focused on polymeric capsules, inorganic porous materials usually exhibit much higher stability concerning the pressure and temperature conditions to be applied. Moreover, due to their tailored porosity the action of the additive could be prolonged in time,^2^ as compared to the application of the bare additive, being able to allow controlled release properties. Some highly porous crystalline materials like zeolites have been employed to change polymer properties, for instance, as an additive to provide flame retardant and antibacterial properties to polymers.^4^ Even though more than 240 zeolite structures are known,^5^ only a few of them are produced and applied commercially, that is, zeolites A and Y, ZSM-5, Beta, and mordenite.^6^

Zeolite Y (a 3D porous material with diameters of 0.74 nm, a supercage of 1.2 nm and smaller cavities with internal diameters of 0.5 nm containing channels of 0.22 nm)^7^ was used as a microcapsule material. Indeed, an encapsulation methodology to fabricate microcapsules of zeolite Y containing α-tocopheryl acetate (commonly known as vitamin E) as an additive was proposed.^2^ These environmentally friendly and biologically nontoxic microcapsules incorporating vitamin E were embedded in fibers of polyamide 6 during its spinning process. As a result, these microcapsules provided the beneficial properties of the encapsulated additive and guaranteed its persistence and long durability.^8^ These fibers are different from other fabrics in which the capsules are commonly applied with superficial treatments in the finishing stages^9^ with the process being limited to the thermal and mechanical characteristics of the materials^10^ and therefore to their useful life because of the wear or a simply laundering.

Compared to other porous materials (activated carbon, zeolites, or silica-based materials), metal–organic frameworks (MOFs) can result in a more efficient way of additive encapsulation due to their affinity with polymeric materials, enabling remarkable loadings (higher than traditional carrier systems) of a large variety of active molecules, such as drugs^11^ or cosmetics,^12^ together with their progressive releases under physiological conditions. MOFs are porous crystalline hybrid materials made of metal ions or clusters coordinated with organic linkers to form 1D, 2D, and 3D crystal lattices.^13^ Apart from their large porosity, high specific surface areas, and excellent chemical and thermal stability,^14^ the possibility of these materials of tuning the pore size, shape, and chemical functionality by modifying the connectivity of the metal ion and the nature of the organic ligands^15^ make them attractive, apart from encapsulation,^16^ for a wide range of applications, such as selective membranes for molecular separation,^17^ adsorption, and storage of gases,^18^ catalysis,^19^ or biomedicine.^11^

Several companies have begun the commercial production of MOFs, mainly ZIF-8 (Basolite Z1200, 2-methylimidazole zinc salt) and HKUST-1 (Basolite C300, known as Cu_3_(BTC)_2_, BTC, benzene-1,3,5-tricarboxylic acid).^20^ Nevertheless, the economic limitations to produce the desired high-quality materials in sufficiently large quantities at low cost make MOF materials unsuitable for industrial scale implementation.^21^ The developments over the past two decades in this area have mainly been based on fundamental studies^22^ obtaining MOFs at lab scale from traditional (mechanochemical or solvothermal) synthesis reactions).^23^ Thus, an exhaustive development from academic research toward potential industrial applications is required.^24,25^

An interesting route to reduce synthesis costs would be replacing current batch methods with a scalable continuous synthesis process.^26^ HKUST-1, MOF-5, MIL-53, IRMOF-3, and UiO-66 have been synthesized by the microfluidic approach but some challenges need to be overcome for further optimization.^27^ High-quality HKUST-1 was also synthesized at a large scale using ethanol as solvent. This resulted in a greener and potentially much more economical process (since the solvent can be recycled).^28^ Microwave heating was also used to produce HKUST-1 in time scales several orders of magnitude faster than by conventional heating. Moreover, high-throughput (HT) methods were employed to prepare porous materials rapidly and cleanly with excellent scalability in particular under the continuous system. This methodology was demonstrated by Reinsch and Stock^26^ to synthesize MIL-53(Al), composed of trivalent metal cations Al^3+^ interconnected through the linker to form a 3D framework with rhombus-shaped 1D channels.^29^ This benchmark MOF combines thermal stability with high porosity^30^ and adsorption selectivity.^31^ This may result from the encapsulation point of view due to the potential biocompatibility of its organic linker (a carboxylate-type ligand, terephthalic acid).^32^ MIL-53 has been also produced at a large scale applying a filter press for filtration and washing steps after crystallization in a conventional reactor. An additional spray dryer might be also integrated into the process to enable efficient drying of the porous product.^33^ The MOF upscaling has also tried to eliminate the use of organic solvents to achieve less toxic and more environmentally friendly processes.^25,34^

Herein, caffeine-MIL-53(Al) microcapsules (CAF@MIL-53(Al)) were upscaled from lab to pilot plant exploring different protocols of MOF activation, solvents, and aluminum sources (Al(NO_3_)_3_·9H_2_O and Al_2_(SO_4_)_3_) with the aim of reducing costs and using processes more environmentally friendly and harmless to health. In addition, also at the large-scaled CAF@MIL-53(Al) microcapsules were introduced during the extrusion process of polyamide to obtain textile fibers with cosmetic properties as an advantageous alternative to usual surface finishes. The presence of caffeine in both CAF@MIL-53(Al) microcapsules and the composite polyamide fibers containing those microcapsules was qualitatively and quantitatively studied by TGA, FTIR, XRD, and GC-MS, while their additive release was monitored by UV–vis spectrophotometer. Fabrics of the composite PA-6 fibers were additionally submitted to washing, scouring, and staining processes and the presence of caffeine was identified after those processes. Finally, to the best of our knowledge this is the first time that a MOF, in particular MIL-53(Al) due to its high porosity and chemical and thermal stability, is applied to produce polyamide PA-6 fibers with encapsulated caffeine presented in this work.

## 2 Experimental Procedure

### 2.1 Synthesis of MOF Capsules

#### Synthesis of MIL-53(Al) in Water

In a typical synthesis, 1Al(NO_3_)_3_·9H_2_O/0.5BDC/80H_2_O molar ratio was used.^29^ Aluminum nitrate nonahydrate (Sigma-Aldrich, ≥98%) and terephthalic acid (BDC, Sigma-Aldrich, 98%) were dispersed in deionized water and placed in a Teflon-lined stainless steel 40 mL autoclave for 3 days at 220 °C. The resulting solid was recovered by centrifugation at 10 000 rpm for 10 min. It was washed with ethanol, separated again by centrifugation, and dried overnight at 65 °C. The product was further activated by several techniques: (i) calcination at different temperatures (330, 350, and 380 °C), (ii) solvent extraction (reflux with methanol at 80 °C), and (iii) interchange with DMF at 150 °C in an autoclave.

#### Synthesis of MIL-53(Al) in Water/Methanol Mixture (1:1 vol.)

The same molar ratio of the reactants for the synthesis in water was used but introducing methanol in the solvent mixture (50/50 (v%) water/methanol) giving to a new synthesis procedure (1Al(NO_3_)_3_·9H_2_O/0.5BDC/40H_2_O/18CH_3_OH). The synthesis temperature was reduced from 220 °C to 180 and 150 °C, while reaction time was also diminished (from 72 to 48, 24, and 12 h) using the same Teflon-lined stainless-steel 40 mL autoclave. By including methanol in the solvent mixture, in addition to reducing the time and temperature of the synthesis, the resulting product was practically activated, as will be shown later.

#### Synthesis of MIL-53(Al) with Aluminum Sulfate Instead of Aluminum Nitrate as Al Source

The aluminum sulfate, SUFAL 8.2, a coagulating and flocculating agent used for potable and wastewater treatments as well as an additive for gluing in the paper industry, was replaced by aluminum nitrate. For that, from the weight percentage of Al_2_O_3_ present in SUFAL 8.2, the necessary amount of reactants was calculated so that the moles of Al present in the reagent was maintained with respect to the synthesis carried out with aluminum nitrate. Different synthesis conditions were tested (temperatures of 150 and 180 °C and times of 12, 24, and 48 h) with the different volume autoclaves: 40 mL, 400 mL and 3.75 L. In addition, a 150 mL autoclave with pressure control was further used to extrapolate these pressure conditions to lab demonstrator level. MIL-53(Al) synthesized by this procedure required proper activation before additive encapsulation.

#### Scaling-up of ML-53(Al)

Previous MIL-53(Al) synthesis at lab scale was reproduced using the same molar composition but with bigger autoclaves, Berghof DAB-3 of 400 mL and Parr 4551 of 3.75 L (useful volume of 3 L). Large-scale synthesis was only done by using water/methanol mixture due to the pressure limitations, which will be addressed later.

#### Synthesis of ZIF-8 and Zeolite Y at a Large Scale

ZIF-8 and zeolite Y were additionally synthesized for materials comparison with MIL-53(Al). According to ref (35), ZIF-8 was prepared upon mixing the reactants at room temperature in the following molar ratio: 1Zn(NO_3_)_2_·6H_2_O/12Im/312.5MeOH/177H_2_O for 2 h at 25 °C. A further step of centrifugation to isolate the ZIF-8 nanoparticles was required. Because of the low particle size of ZIF-8 (about 100 nm) the use of a centrifuge was more efficient rather than filtration. Zeolite Y was produced at IQE, S.A., under the trademark SIOLITE with SiO_2_/Al_2_O_3_ 4.8. Before encapsulation, a pretreatment stage was required (250 °C in an oven for 24 h) to activate the zeolite surface and pores to facilitate the sorption process.

#### Caffeine Encapsulation

The procedure for caffeine encapsulation in MIL-53(Al) capsules consists of contacting double amount by weight of caffeine than MOF (200% caffeine) in water in a closed container at 80 °C for 24 h. In a typical lab-scale encapsulation 0.3 g of MOF and 0.6 g of caffeine in 60 g of water are used. For large-scale encapsulation, all of the above quantities are multiplied by 200. Note that the same encapsulation process was followed for ZIF-8 and zeolite Y microporous materials. Once filtered (CAF@MIL-53(Al) and CAF@zeolite Y) or centrifuged (CAF@ZIF-8), the capsules were dried at room temperature and subsequently submitted to a thermal treatment stage (80 °C for 12 h).

### 2.2 Delivery Measurements and Extraction of Caffeine

#### Additive Delivery

Delivery measurements of caffeine in the capsules were carried out at room temperature (about 25 °C) in a V-670 Jasco UV–vis spectrophotometer. The measurements were taken at the maximum absorption wavelength of the caffeine molecule (272.5 nm). For that, suspensions of 10 mg of CAF@MIL-53(Al) in 100 mL of deionized water were prepared and the concentration of the caffeine released over time was determined by using a calibration curve.

#### Extraction in a Condenser

The caffeine encapsulated in the MOF and zeolite microcapsules was extracted under ethanol reflux for 12 h. In the case of the composite polyamide fabrics, the extraction was carried out under ethanol reflux for 24 h. Caffeine was analyzed by GC/MS by diluting 1 mL of the solution 25 times in ethanol. For that, an Agilent 6850 gas chromatograph with a 5975C VL MSD mass spectrometric detector was used. Caffeine was separated by means of an HP-5MS capillary column, 30 m × 0.25 mm I.D., and 0.25 μm phase thickness. The operation was done in the electron impact mode (EI, 70 eV) and *m*/*z* 194 ion was selected for monitoring. Caffeine was identified by direct comparison with caffeine standard on the basis of the retention time and mass spectral ion ratios in the corresponding dilutions.

### 2.3 Preparation of Composite Polyamide-6 Fibers Containing Caffeine-Encapsulated Microcapsules

#### Industrial Spinning Process

The caffeine-encapsulated particles were supplied to the industrial spinning process at Nurel S.A. with the aim of producing the composite polyamide-6 fibers in fabrics with cosmetic properties. For that, the following different concentrations of capsules were used: 0.28 wt % of zeolite Y, 0.6 wt % of ZIF-8, and 0.35, 0.45, and 0.70 wt % of MIL-53(Al).

Nurel POY (partially oriented yarn) spinning line is equipped with a single screw extruder model EM 45FO with a 45 mm screw diameter and an *L*/*D* ratio of 24. The production capacity of the machine is variable up to 20 kg/h. Once the polymer melts into the extruder it goes through two spinning pumps, dividing the flow into six smaller ones and conducing the polymer to the spin packs. Six different fibers are cooled down and winded using a Barmag winder at different speeds (up to 5000 m/min). This technology delivers six yarn bobbins. A simple scheme of the spinning process can be seen in Figure S1. More information on the fabrication process of the composite polyamide fibers with cosmetic properties can be found in ref (8).

#### Washing, Scouring, and Staining Procedures in Fabrics

The presence of caffeine in the fabrics made from the fibers was verified by GC/MS after several common textile procedures. In all of them, the composite PA-6 fabrics were put in contact with water at different time and temperature conditions: (a) long washing machine (30 °C, 90 min, and neutral soap), (b) scouring (40 °C, minimum of 10 min, liquid detergent (0.5 g/L), and (c) staining in blue (100 °C for 1 h, Turquoise M-5G acid dye at 1.5% in deionized water). The staining procedure of the fabrics was done by using a horizontal autoclave. The last step for the three procedures consists of an abundant water rinsing followed by centrifugation and drying.

### 2.4 Characterization Techniques

#### Characterization of the Capsules

Powder X-ray diffraction (XRD) was analyzed using a D-Max Rigaku X-ray diffractometer with a copper anode and a graphite monochromator to select CuKα radiation (λ = 1.5418 Å). Data were collected in the 2.5–40° 2θ range with a scanning rate of 0.03°/s. Thermogravimetric analyses (TGA) were performed using Mettler Toledo TGA/SDTA 851^e^ equipment. Samples placed in 70 μL alumina pans were heated in N_2_ flow up to 750 °C with a heating rate of 10 °C/min. BET specific surface area was measured with a Micromeritics TriStar 3000 with a previous degasification at 150 °C for 5 h. The Fourier transformed infrared spectroscopy (FTIR) absorption spectra were acquired at room temperature with an Iraffinity Shimadzu spectrophotometer. Spectra of the samples corresponded to 30 scans at a resolution of 4 cm^–1^ using the KBr pellet technique. The scanning electron microscopy (SEM) images were taken with an INSPECT F50 microscope (Thermofisher) at 2–15 kV after precoating the samples with palladium.

#### Characterization of the Composite Polyamide Fibers

The tensile testing (tenacity and elongation at break) of the polyamide fibers was done using a Model STATIMAT ME+ testing instrument from TEXTECHNO according to NA-EPA-051. All of the tests were performed at standardized conditions of 21 °C (±1 °C) and 65% (±2%) relative humidity. Pneumatic yarn grips were used for these tests with the effective gauge length set at 300 mm and a crosshead speed of 300 m/min. The study of the morphology of the composite polyamide fibers was carried out by SEM. For this purpose, several sections of the fabrics (as prepared, after a long washing machine, and after scouring/staining) were freeze-fracturing after immersion in liquid N_2_. In addition, a piece of fabric containing 0.70 wt % of MIL-53(Al) was embedded in EMBed812 epoxy resin and polymerized at 60 °C for 24 h. Afterward, 1.5 μm thick sections were obtained using a diamond knife (Histo 45°, Diatome) and the ultramicrotome Leica EM UC7. Sections of about 1.0 × 0.7 mm^2^ were picked from the water bath and deposited on a pin stub with carbon tape and coated with 14 nm of palladium or 20 nm of carbon. The pin was previously glow discharged (30 s, 15 mA) to promote the deposition of flat sections.

## 3 Results

### 3.1 Selection of MIL-53(Al) Material for Upscaling and Caffeine Encapsulation

The synthesis of the MOF and the encapsulation process need to be defined and optimized for subsequent scaling-up. Among porous Al-carboxylate series, MIL-53(Al) is attracting considerable attention for scaling-up and further application for additive encapsulation to prepare functionalized textile fibers. MIL-53 (Al) has been chosen here due to several advantages over other MOF or purely inorganic materials:

#### Economic and Environmentally Friendly Reactants

Aluminum-based MOFs are commercially available and are used for tests in large-scale applications.^21,26,28,33,36^ The low toxicity plays an important role in the handling of the materials and for their application.^26^ To synthesize MIL-53(Al), aluminum salt and a terephthalate linker is needed: (a) aluminum nitrate can be used following the original MIL-53 recipe^29^ but the employment of another salt, that is, aluminum sulfate, which IQE S.A. manufactured as SUFAL 8.2, is of interest to be explored to prevent the safety hazard caused by nitrates^36^ in addition to its more reduced price; (b) benzene-1,4-dicarboxylate linker, commonly known as BDC (coming from terephthalic acid, TPA). In fact, TPA is a cheap material that was also employed at a large scale by Nurel S.A. in its industrial production process as a precursor to the polyester PET and polybutylene terephthalate PBT for application in the technical polymer. Green solvents, such as water or a mixture of water and methanol, are also used for the synthesis.

#### Scale-up Synthesis Facility

For large-scale production of a porous material, the availability and purity of the chemicals, raw material costs, toxicity of reagents, and safety play an important role. When synthesizing MOFs from diluted solutions, large material costs generally are derived from the organic solvent employed, which also concerns filtration and washing stages at the industrial scale. This is the reason why water is always the most attractive solvent for industrial scale. Moreover, applying high temperatures (>200 °C) is often necessary for the synthesis of Al-based MOFs, which leads to high autogenous pressure in the reactor (>20 bar).^26^ Together with the price of the organic solvents, the use of these extreme synthesis conditions are important steps in the transfer of the synthesis protocol from laboratory to technical scale at the industry. Specifically, the synthesis procedure of MIL-53(Al) can be up-scaled by using different reactor volumes. Some parameters such as molar ratio of reactants, solvents used, time, and temperature of reaction (that directly affect the pressures reached in the different volume reactors) or activation treatments (to enhance the encapsulation capacity) need to be optimized for a successful MIL-53(Al) powder production at various levels of scaling-up.

#### Porous Flexible Structure with Breathing Behavior

**Figure 1:**
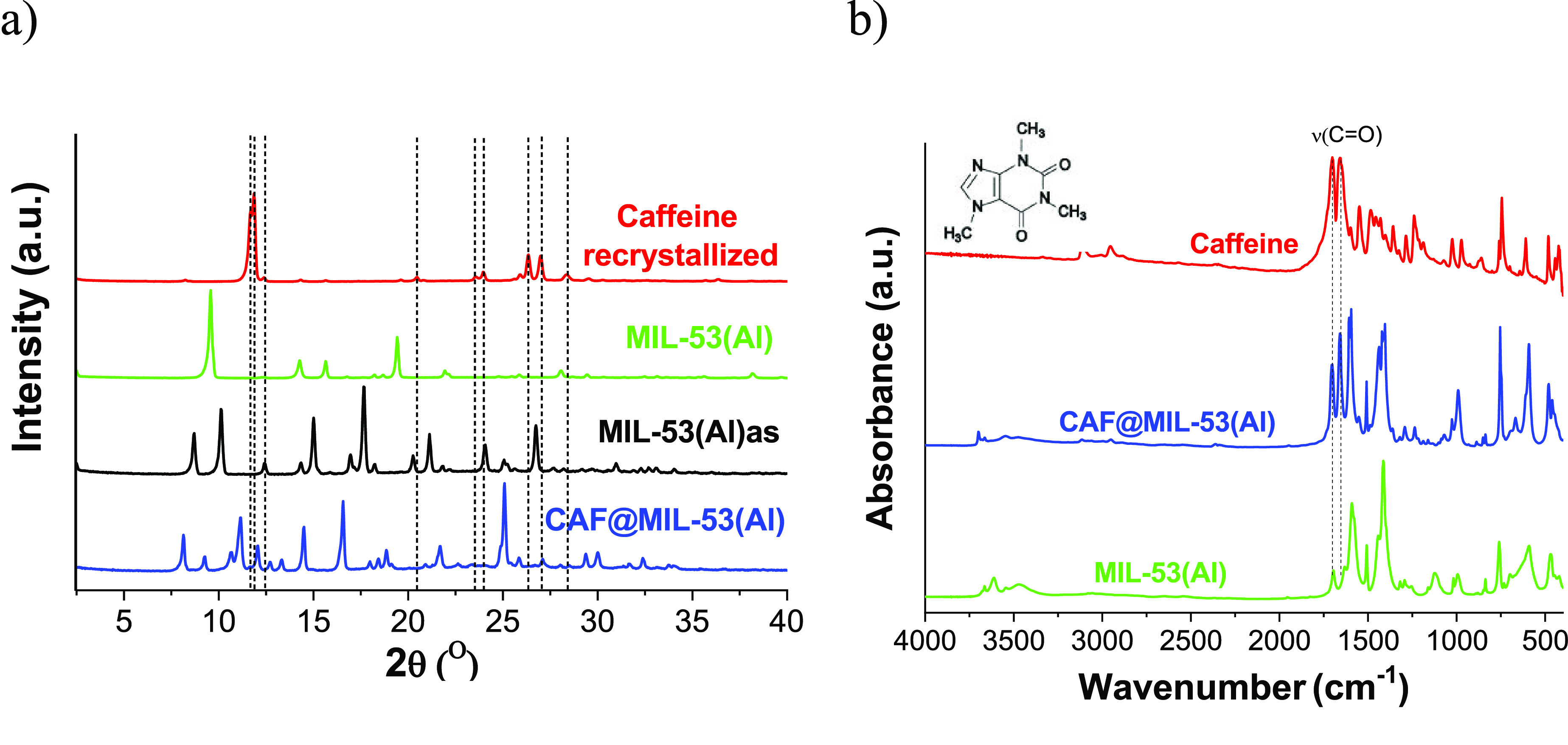
CAF@MIL-53(Al) sample (encapsulation MOF/caffeine weight ratio of 1:2), together with as-synthesized in water MIL-53(Al)as, activated MIL-53(Al), and pure caffeine recrystallized under the conditions of synthesis of MIL-53(Al) for comparison. (a) XRD patterns, (b) FTIR analysis.

MIL-53(Al) has the property to adapt its porosity due to a pore widening which can vary in the range of 8.5 × 8.5 Å (high temperature (*ht*) form) and 2.6 × 13.6 Å (low temperature (*lt*) form), according to the size and shape of the guest molecule.^29,37^ For instance, this tunable porosity of MIL-53(Al), controlled by the interactions between the encapsulated molecules and the pore wall of the MOF structure, led to an exceptionally long and progressive release (up to 3 weeks) of 20 wt % encapsulated ibuprofen.^38^ Concerning the reference additive studied in this work, caffeine (C_8_H_10_N_4_O_2_, boiling point 178 °C, and highly water-soluble, see structure in Figure 1b), it has been successfully encapsulated in ZIF-8 (CAF@ZIF-8) by in situ one-step-demonstrating high guest loading (about 28 wt %) and controlled additive release (during 27 days).^35^

#### Exceptional Additive Encapsulation in MOF Materials and Biocompatibility

The liporeductor cosmetic caffeine molecule has been used as an encapsulation agent in a large range of biocompatible porous metal carboxylates MOFs.^11,38^ Compared to inorganic capsules the high and regular porosity of MOFs combined with their amphiphilic internal microenvironment may allow the achievement of record additive loadings and controlled releases under simulated physiological conditions (phosphate buffer solution pH = 7.4 or distilled water pH = 6.3, 37 °C).^39^ Indeed, by using MOFs the advantages of lightness, flexibility, porosity, chemical affinity, and adsorption capacity could mean a reduction of the number of microcapsules needed for fiber additivation, maintaining or even increasing the additive encapsulation content in a more efficient way. For instance, MIL-100(Fe), MIL-53(Fe) and UiO-66 appeared as very promising carriers for caffeine encapsulation with very interesting cosmetic play loads (49.5, 29.2, and 22.4 wt %, respectively) with progressing releases within 24 h.^12^

#### Proper MOF-Polyamide Fiber Compatibility

Because of the organic–inorganic character of the porous material, the interaction of terephthalic acid with the polyamide 6 fibers will become enhanced.

#### High Thermal Stability

High thermal stability represents the determinant factor for certain successful encapsulation treatments. In the case that concerns us, the capsule with the additive trapped must be stable under the spinning conditions to produce the polyamide fibers and the fabric (about 260 °C). Generally, Al-carboxylates exhibit extraordinary thermal stability up to about 450 °C.^40^ Specifically, MIL-53(Al) is a stable MOF up to just over 400 °C (see Figure S2a), and it can be thermally activated at 380 °C (see below). A thermal stabilization of the additive caffeine is expected through encapsulation related to its adsorption on the MIL-53(Al) porosity and not to their mere external impregnation.

### 3.2 Synthesis of MIL-53(Al) with Al(NO_3_)_3_·9H_2_O: The Study of the Activation Treatment and Liquid Phase Caffeine Encapsulation

#### Synthesis in Water: From Lab Scale to Pilot Plant

TGA analysis (Figure S2a) shows that BDC molecules (about 27.5%, weight loss between 350 and 500 °C) are trapped in the structure of MIL-53(Al)as (as-synthesized MIL-53(Al)). MIL-53(Al)as required an activation treatment to evacuate the nonreacted trapped BDC molecules. Diverse activation procedures were considered: calcination and solvent extraction. Table 1 shows the amount of nonreacted ligand for each activation condition. In this material, high temperatures are required to open and free the pores. Thus, temperatures of 330, 350, and 380 °C were explored with temperatures always lower than the stability of the MOF to ensure that the structure is maintained. The best condition corresponded to 380 °C obtaining a complete activation of the material while preserving its structure (see below). Note that in this sample there was a loss of adsorbed water (weight loss below 100 °C). Another method more energetically sustainable would be liquid phase extraction. However, this type of procedure is associated with the use of low environmentally friendly solvents. Heating the MOF at 150 °C in an autoclave with DMF also produced an activated MIL material (see Figure S2b, with only 2.3 wt % of BDC molecules trapped). As it is well-known, DMF is a toxic and teratogenic reagent that requires special safety actions, especially when applied at pilot plant scales.^37^ Methanol reflux was also used leaving a large amount of linker trapped in the structure (17.4 wt %, see Table 1). DMF and methanol were therefore not recommendable for large-scale synthesis with calcination being the only option.

**Table 1:** Properties of MIL-53(Al) Synthesized at Lab Scale and Pilot Plant^a^

	synthesis in H_2_O (220 °C, 72h)		synthesis in H_2_O/MeOH (150 °C, 12h)	
upscaling size	lab scale^b^		lab scale	pilot plant
MIL-53(Al)as (trapped BDC, wt %)	27.5 ± 1.9		9.9 ± 1.1	13.3 ± 0.7
	needs activation		practically activated^c^	
MIL-53(Al) activation treatments (trapped BDC, wt %)	MeOH wash (80 °C)	17.4		
	DMF treatment (150 °C)	2.3		
	calcination 380 °C	complete activation		
Caffeine encapsulation (wt %)^d^	38.0^e^		36.5 ± 0.5	34.6 ± 1.7^f^

a Linker content in MIL-53(Al)as (as-synthesized) in water and water/methanol mixture and percentage of caffeine encapsulated. All of the samples were characterized by TGA. Standard deviations were calculated from the averaging of at least three batch syntheses.

b Synthesis in H_2_O at pilot plant scale was not successful due to the breakage of the rupture disk.

c No activation was required before caffeine encapsulation.

d Caffeine encapsulation is the percentage of caffeine in the capsules = CAF(g)/CAF@MIL-53(Al)(g) × 100, excluding residues of ligand and solvents (i.e., losses below about 250 °C).

e Obtained from MIL-53(Al) activated by DMF treatment.

f At least 10 encapsulations were done at pilot plant scale to obtain the amount required for polyamide spinning processes.

TGA was further useful to calculate the caffeine encapsulation yield. When molecules are encapsulated they show a thermal stabilization, something that does not happen when the additive is impregnating the external surface of the porous particles, not penetrating the MOF structure. Figure S2b (inset) shows a clear displacement of the caffeine peak from about 265 to 325 °C consistent with the incorporation of caffeine in the MIL-53(Al) structure.

XRD is a qualitative tool for monitoring the effective encapsulation of the additives in which the intensity of the guest molecules decreases upon its contact with the MOF due to the adsorption in the MOF structure.^41^Figure 1a shows the XRD patterns corresponding to MIL-53(Al), as-synthesized (as) and activated at 380 °C, together with caffeine which has been recrystallized under the conditions of synthesis of MIL-53(Al) and caffeine encapsulated MIL-53(Al) (CAF@MIL-53(Al)) for proper comparison. XRD of CAF@MIL-53(Al) shows a crystalline structure that changes concerning the activated form given the flexibility of the MIL-53 structure when molecules are occluded in its pores with different pore opening, and highlights the absence of the characteristic recrystallized caffeine peaks consistent with its encapsulation.^41^ To further corroborate the presence of caffeine in the samples without losing their chemical integrity, an FTIR analysis was carried out. Figure 1b shows displacements in the ν(C=O) band of the encapsulated sample with respect to pure caffeine (about 1658 and 1702 cm^–1^), as well as the presence of caffeine peaks around 2950 and 3100 cm^–1^, corresponding to the vibration of the C–H bands.

Before going to a MIL-53(Al) pilot plant scale, the synthesis in an autoclave of 400 mL was carried out as an intermediate dimension between the synthesis at lab-scale (where one usually works with a 40 mL-sized autoclave) and the lab demonstrator (autoclave of 3.75 L). For that, the same molar ratio of reactants was used but multiplying the amounts by 8 as compared to the lab-scale synthesis (see Table 2), resulting in reproducible TGA and XRD results. With this premise, the synthesis was up-scaled to the pilot plant autoclave. The amounts of reactants were then multiplied by 10 (×80 considering the 40 mL autoclave) and the reaction was set at 220 °C for 3 days. Two nonsuccessful attempts occurred. In both, the breakage of the reactor rupture disk happened during the first 24 h of reaction. It is speculated that during the synthesis process nitric acid was produced, which generated hydrogen (increasing pressure) and at the same time corroded the metal of the rupture disk. This effect then caused the maximum pressure that it can support to decrease, breaking the rupture disk below the theoretical 120 bar for a temperature of 220 °C. Gaab et al.^40^ also observed a similar behavior for the synthesis of various Al-MIL MOFs, leading to a high autogenous pressure in the reactor when the synthesis is produced at temperatures higher than 200 °C. For that reason, a reduced temperature was considered from now on.

**Table 2:** Synthesis Conditions and Amount of MIL-53(Al) Obtained by Using Nitrate and Sulfate (SUFAL 8.2) as an Aluminum Source at Four Reactor Volumes^a^

		Al-nitrate		Al-sulfate		
reactor volume (mL)	solvent volume (mL)	synthesis conditions	average MOF mass (g)	synthesis conditions (synthesis no.)	*P*_max_ (bar)	MOF mass (g)
40	10 mL H_2_O + 10 mL MeOH	150 °C, 12 h	1.5 ± 0.1	150, 24 h		1.5
150	(×3)			(1) 150 °C, 24 h	14	2.5
				(2) 150 °C, 48 h	19	2.8
				(3) 180 °C, 24 h	33	3.0
400	(×8)	150 °C, 12h	12.1 ± 2.1^b^	150, 24 h		8.6
3750	(×80)	150 °C, 12h	161 ± 15^c^	(1) 180 °C, 12 h	46	108
				(2) 150 °C, 12 h	12	83
				(3) 150 °C, 24 h	20	86

a All of the syntheses were carried out in a water/methanol mixture as solvent. Maximum pressures recorded during the synthesis with 150 mL and 3.75 L volume reactors for Al-sulfate source were included. Standard deviations were generally calculated from the averaging of at least three syntheses in each reactor volume.

b This was calculated as the average of 12 syntheses.

c This was calculated as the average of 5 syntheses.

##### Synthesis in Water/Methanol Mixture: Lab-Scale Synthesis

Because of the inconvenience of MOF activation, new synthesis conditions were studied on a laboratory scale by introducing methanol together with the water as solvent. It is also necessary to mention the environment and safety problems that could arise when activating large batches of MOF by calcination with the need to find a synthesis procedure compatible with up-scaling limiting conditions.

Even though in the first studies the high temperature (220 °C) and time (72 h) were followed from literature,^29^ some attempts to diminish temperature up to 150 °C and reduce time up to 12 h were made. Figure S3 shows the TGA of the materials prepared at 150 °C under water/methanol mixture at different reaction times (72, 48, 24, and 12 h) together with the TGA of MIL-53(Al)as (synthesized only in water) for comparison. No significant differences were depicted among the various reaction times, achieving at the lowest reaction time less than 10% of nonreacted BDC molecules (Table 1). Thus, when the synthesis is carried out in a water/methanol mixture at 150 °C for 12 h the MOF is activated to a considerable degree and does not require further treatment. Therefore, the amount of BDC present in the resulting material may not interfere in the encapsulation process, as occurred previously with the MOF synthesized in water that needs additional activation. In addition, Figure S4a shows that the encapsulation of caffeine in MIL-53(Al)as_H_2_O without activation was not carried out properly since the material after the encapsulation process presents practically the same thermogram as the starting material. Nevertheless, when MIL-53(Al)as_H_2_O/MeOH is prepared for 12 h a clear difference between the encapsulated sample and the starting material is noted (Figure S4b) with this difference being the caffeine that has been incorporated in the material. This encapsulated caffeine was degraded at temperatures higher than those corresponding to the pure additive. This fact indicates greater stability and resistance of the additive to the temperatures of the yarn manufacturing process, where up to 260 °C can be reached for short times.

Figure S5 shows a comparison of the X-ray diffraction of MIL-53(Al) synthesized under the different conditions: in water and water/methanol mixture solvent and caffeine encapsulation taking both, the former, calcined at 380 °C and, the latter, as-synthesized capsules. CAF@MIL-53 XRD peaks do not coincide with the main peaks of recrystallized caffeine. Some changes can be depicted with respect to the activated form due to the flexibility of the MIL-53 structure when the additive is occluded in its pores, as previously reported.^12^ By TGA, it was also corroborated that encapsulation takes place without the necessity of an extra activation for the synthesis done in water/methanol mixture. Activated MIL-53(Al)_H_2_O and MIL-53(Al)_H_2_O/MeOH were also observed by SEM, and Figure S6 shows that MIL-53(Al) synthesized in the water/methanol mixture is more homogeneous in size than when it is prepared in water. The more uniform MOF particle size could be due to the higher solubility of the ligand in the organic solvent giving rise to more homogeneous nucleation and crystal growth processes. In addition, the size of the crystals is highly reduced to about 0.5 μm in comparison with particles synthesized in water of about 3 μm that can reach dimensions up to 10 μm.

##### Synthesis in Water/Methanol Mixture: Pilot Plant Synthesis

**Figure 2:**
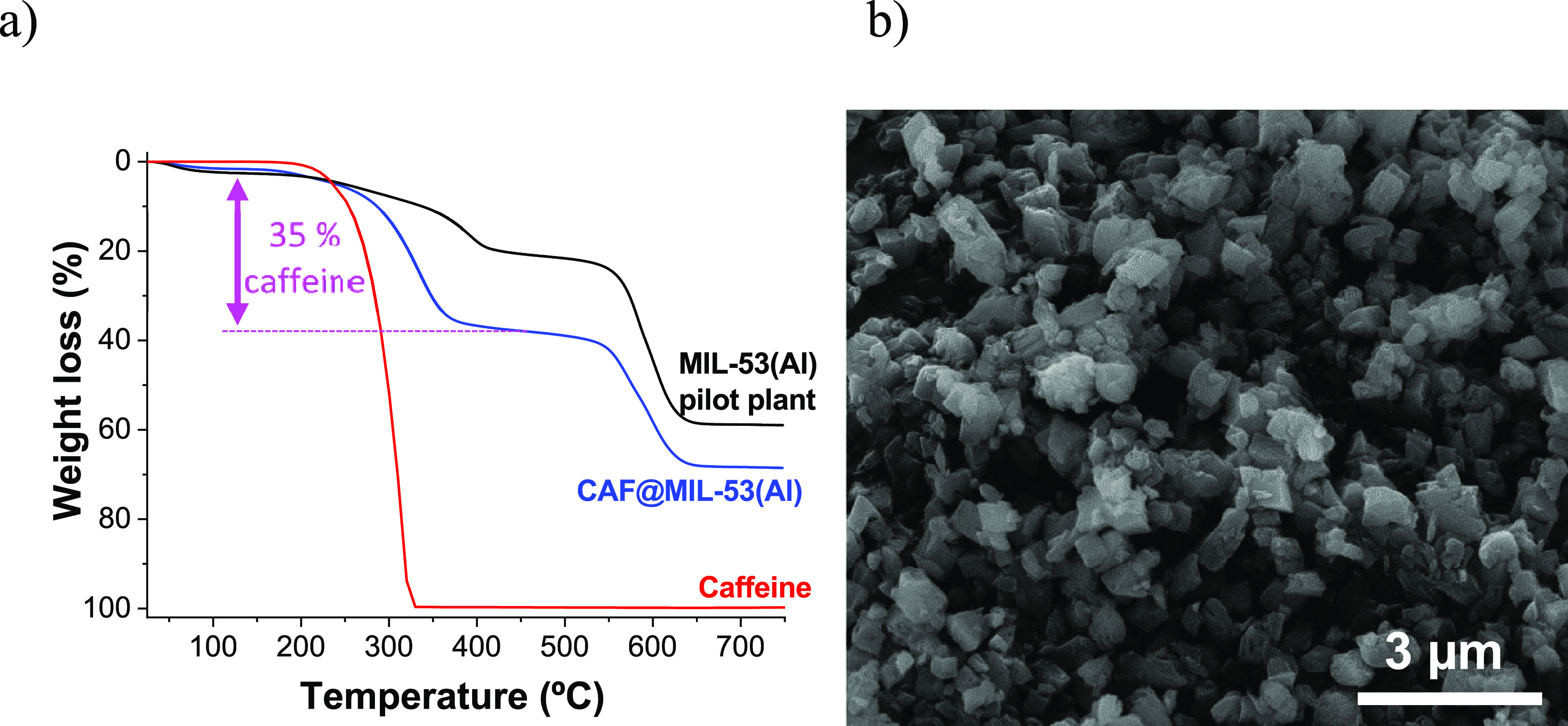
MIL-53(Al) synthesis with a water/methanol mixture solvent at pilot plant scale: (a) TGA, (b) SEM. TGA of CAF@MIL-53(Al) is included inferring 35 wt % of caffeine encapsulation.

At least five syntheses were carried out for each level of scaling-up at a temperature of 150 °C for 12 h. Table 2 shows the amounts of MIL-53(Al) powder obtained for the different scaling-up syntheses. Even though the incorporated terephthalic acid trapped in the structure is of about 13.3 wt % (see Table 1), this fact does not practically affect the caffeine encapsulation being still in the range of 35–37 wt % (see Figure 2a and Figure S4b for the TGA of pilot plant and laboratory-scale syntheses, respectively), as will be also corroborated later by GC-MS. At this point, it is highlighted that when the synthesis is done with water at lab scale (with a further activation process) the encapsulated caffeine was a little bit higher (about 38 wt %) (Table 1). However, such a small amount of caffeine encapsulation enhancement, about 2 wt %, does not compensate for the requirement of MOF activation with a high energy consumption for larger scale syntheses.

MIL-53(Al) at large-scale production samples were also analyzed by nitrogen adsorption. An average BET specific surface area value of 905 ± 122 m^2^/g was achieved based on five different syntheses. As a reference, 1140 m^2^/g was previously achieved in lab-scale synthesis of MIL-53(Al), which reduced substantially to 334 m^2^/g when caffeine was encapsulated filling the MOF pores.^41^Figure 2b shows the MIL-53(Al) powder produced at a large scale. More rounded particles compared to the ones obtained at lab-scale (see Figure S6b) were depicted in addition to being slightly larger in size (particles close to 1 μm). This size is suitable for the dispersion of the particles into the polymeric fibers without falling within the classification of nanoparticles whose use in textiles generates some controversy.^42^

At least 10 encapsulations (60 g of MOF/120 g of caffeine) were done obtaining an average amount of material in every process of 99 ± 12 g of CAF@MIL-53(Al). With the sum of all the encapsulations, about 1 kg of CAF@MIL-53(Al) was produced and used for two subsequent spinning processes.

### 3.3 Synthesis of MIL-53(Al) with Al_2_(SO_4_)_3_ (SUFAL 8.2): From Lab Scale to Pilot Plant

To reduce costs at a large scale level, aluminum nitrate was substituted by cheaper Al_2_(SO_4_)_3_, commercialized as SUFAL 8.2. Table 2 shows the syntheses carried out with the MOF weights obtained for each reactor volume, 40 mL, 150 mL, 400 mL, and 3.75 L, in comparison with those prepared with aluminum nitrate.

Figure S7 depicts that the XRD patterns of the synthesis produced with SUFAL 8.2 are very similar but not identical. Given the MIL-53 structure flexibility, the observed dissimilarities can be due to some differences in the molecules occluded in the porous structure compared to the MIL-53(Al) synthesized with aluminum nitrate.

Given the feasibility of synthesizing the MOF with BDC carboxylate ligand through this procedure with SUFAL 8.2, before taking it to the large scale production new syntheses were carried out using a 150 mL reactor volume with which it was possible to measure the pressure. This way, the evolution of the pressure with reaction time was studied, obtaining at 24 h of synthesis a maximum value of 33 bar (at a temperature of 180 °C) versus 14 bar (carried out at 150 °C, whereas 19 bar was achieved when the reaction time was augmented to 48 h). These pressures, even if they are above the expected water vapor pressure at the working temperature, are acceptable and did not compromise the reactor integrity. Reinsch and Stock^26^ also obtained several Al-based MOFs under mild reaction conditions by using Al_2_(SO_4_)_3_·18H_2_O as metal salt. In such study, in the solvothermal system Al_2_(SO_4_)_3_·18H_2_O/poly(carboxylic acid)/H_2_O/DMF with H_2_O as the major solvent, due to the low reaction temperatures employed (125–145 °C) the autogenous pressure inside the reactor did not exceed 5 bar. They also observed that by using aluminum sulfate instead of nitrate or chloride salts, the safety hazard caused by nitrates and the set of corrosion problems originated by chlorides were prevented.

**Figure 3:**
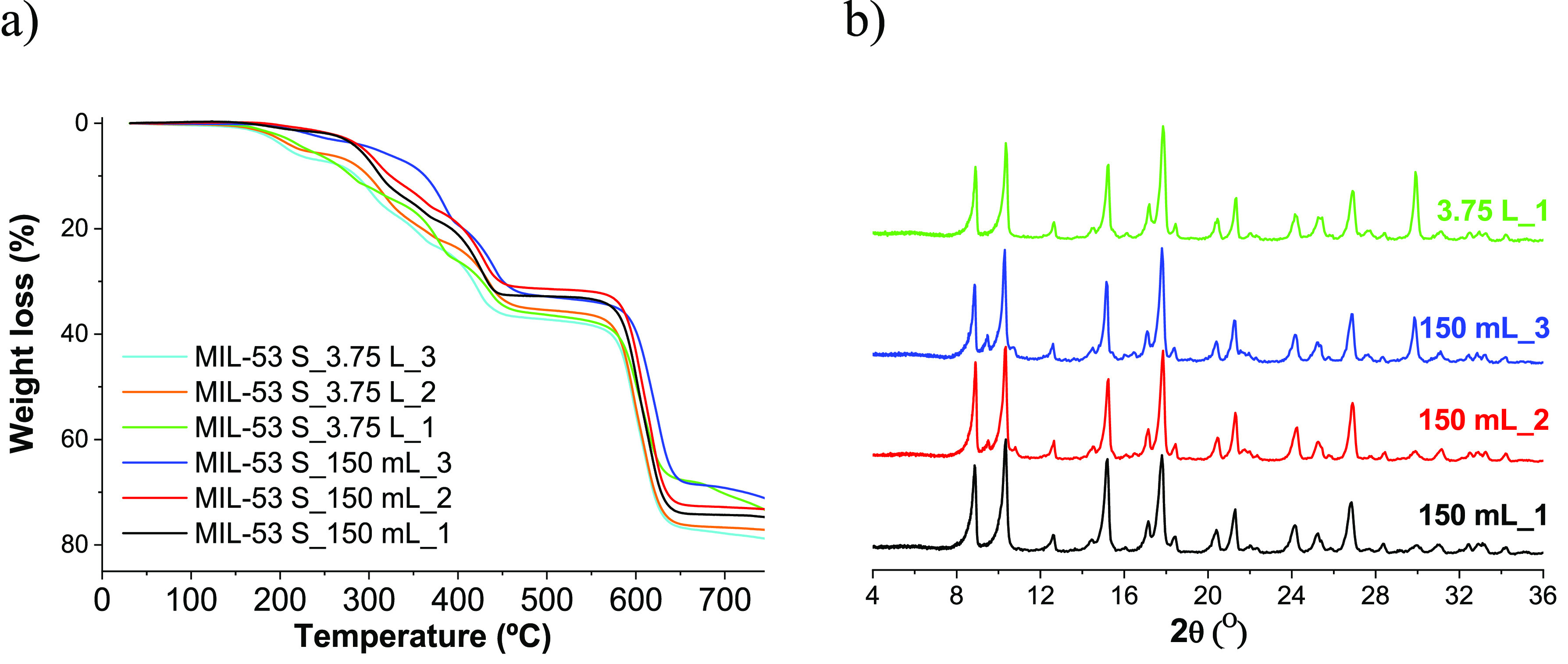
MIL-53(Al) synthesized with SUFAL® 8.2 at the lab and large scales. Synthesis at different conditions of temperature and times specified in Table 2: (a) TGA curves, (b) XRD patterns.

The syntheses prepared in the 150 mL and 3.75 L autoclaves were characterized by TGA and XRD, and the results are plotted in Figure 3. No significant differences between the two-volume reactors are depicted from TGA curves (Figure 3a) and XRD patterns (Figure 3b).

Table 2 shows that the pressure values are higher when the synthesis is done at the pilot plant level. This increase of pressure in the reactor may be due in part to the fact that it does not have a Teflon lining, as normally used at a laboratory scale. Therefore, some parts of the reagents may react with the stainless steel of the reactor producing hydrogen gas as a consequence of the oxidation of the metal.

**Figure 4:**
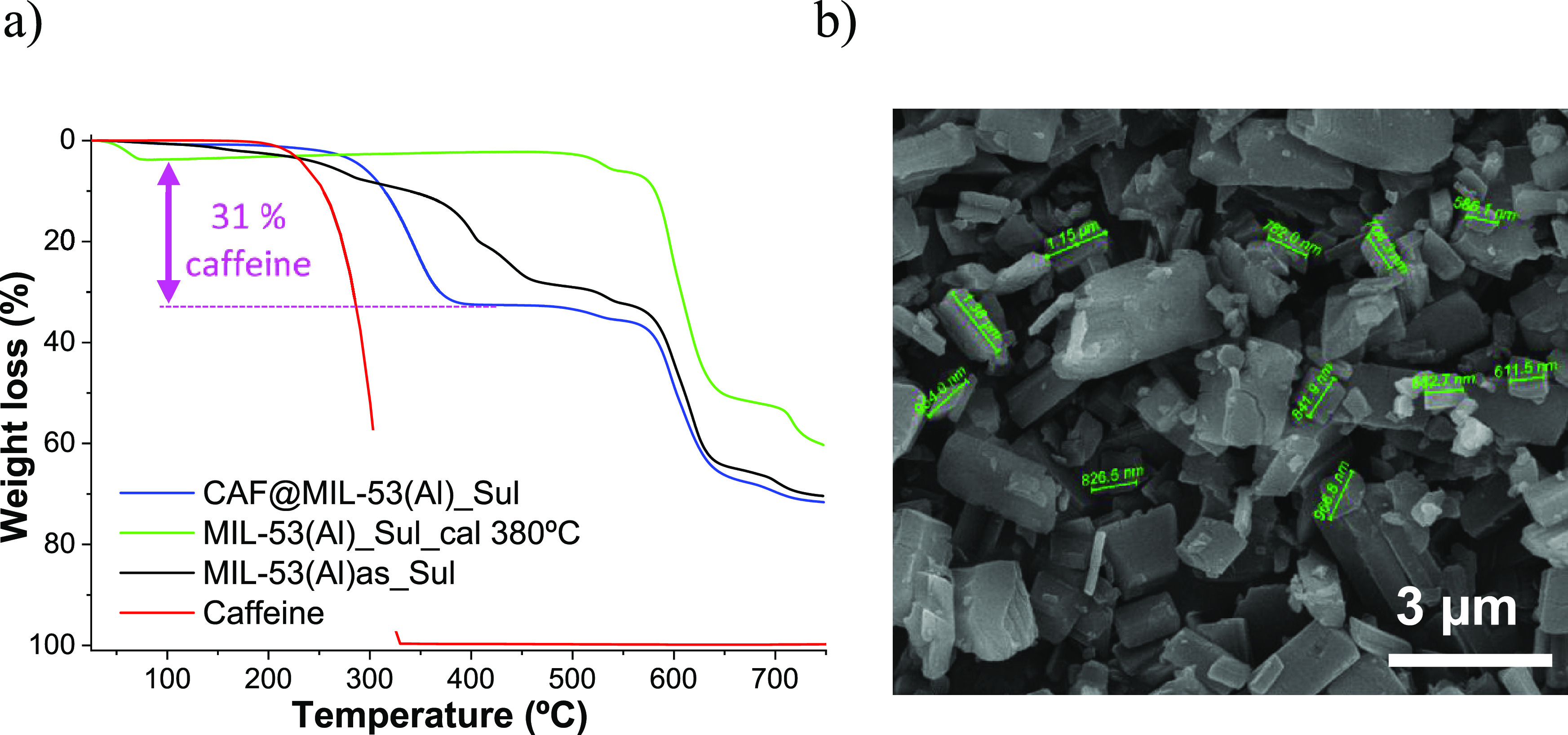
MIL-53(Al) synthesized with SUFAL 8.2 with water/methanol solvent mixture at pilot plant scale. (a) TGA. (b) SEM. TGA of CAF@MIL-53(Al) SUFAL® 8.2 is included inferring 31 wt % of caffeine encapsulation.

In addition, a greater amount of powder was obtained when the reaction was carried out at 180 °C, for both 150 mL and 3.75 L, while the maximum pressure in the reactor was 46 bar, at the highest reactor volume. This pressure is still affordable, being in the range of the one obtained by using aluminum nitrate when the syntheses both with nitrate and sulfate are prepared by using water/methanol mixture as solvent. The TGA curve in Figure 4a depicts a large amount of nonreacted carboxylate ligand (25–30 wt %, corresponding to the step at about 200 °C) that could interfere in the encapsulation process. Unlike the synthesis of MIL-53(Al) with Al(NO_3_)_3_ which generated an activated material, the synthesis carried out with SUFAL 8.2 required activation by calcination at 380 °C for 24 h to remove the terephthalic acid molecules from the pores. Otherwise, such molecules could prevent additive adsorption. For that reason, a trade-off between the cost of Al salt and the effort needed to activate the MOF was considered, and only MIL-53(Al) synthesized with nitrate salt was used to encapsulate caffeine and formulate composite polyamide fibers (see below). It should be noted that the yield of MIL-53 synthesis with respect to the limiting reagent is greater than 50% without clearly influencing the aluminum source used.

As inferred from Figure 4a, 31 wt % of caffeine was encapsulated in MIL-53(Al) synthesized with SUFAL 8.2 at large scale after calcination of the material at 380 °C. Figure 4b reveals MIL-53(Al) SUFAL 8.2 particles of about 1–3 μm but with less homogeneity in sizes as compared to the MIL-53(Al) particles obtained from Al nitrate in Figure 2b.

### 3.4 Caffeine Content in MIL-53(Al) Capsules

#### Comparison with Another Large-Scale Synthesis of Zeolite Y and ZIF-8

**Figure 5:**
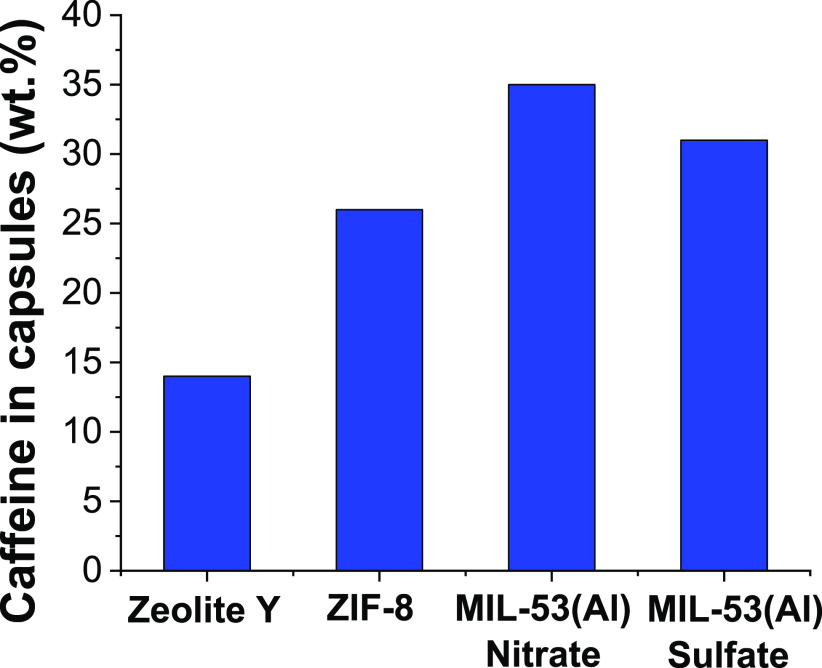
Percentages by weight of caffeine encapsulated in the different capsules synthesized at large scale.

As revealed previously, MIL-53(Al) was synthesized at a large scale using two aluminum sources, nitrate and sulfate, achieving 35 and 31 wt % of encapsulated caffeine, respectively. These values are plotted in Figure 5 in comparison with those achieved in this work also at large scale synthesis based on the porous materials zeolite Y and ZIF-8, highlighting the highest caffeine encapsulation in MIL-53(Al) material from nitrate salt. Figure S8a shows thermograms of caffeine and zeolite Y, which allow us to estimate that the encapsulated caffeine was 14 wt %. The recipe of CAF@ZIF-8 ex situ (ZIF is first synthesized and then caffeine is encapsulated)^33^ was scaled up from about 0.5 g (commonly obtained at lab scale) to 500 g (at lab demonstrator). Figure S8b shows the TGA curve corresponding to CAF@ZIF-8 prepared at a large scale where a weight loss of about 26 wt % assigned to caffeine can be obtained.

#### Study of Controlled Delivery

According to previous measurements by TGA and corroborated also by GC/MS, up to 38 wt % caffeine was found in CAF@MIL-53(Al)_H_2_O/MeOH at lab-scale synthesis. In addition, these microcapsules were submitted at 80 °C for 12 h, reaching a caffeine percentage of 38.9 by UV–vis, verifying therefore the good agreement between the different characterization techniques.

**Figure 6:**
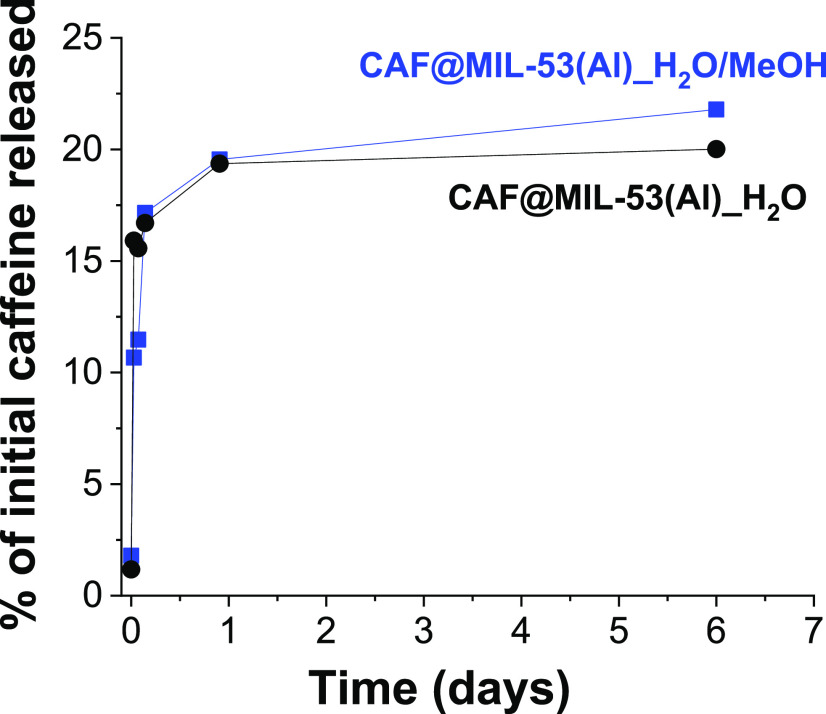
Caffeine delivery of CAF@MIL-53(Al) over time at 25 °C tested by UV–vis.

Figure 6 shows the controlled delivery over time of caffeine for CAF@MIL-53(Al)_H_2_O/MeOH at 25 °C. For that, the microcapsules were suspended in an aqueous medium under stirring and several aliquots were taken and analyzed by UV–vis. During the first hours, the release of caffeine is very fast, but after 1 day and up to 6 days the release slows down, reaching only 22% of the initial caffeine released. The same procedure was done with CAF@MIL-53(Al)_H_2_O analyzing the 20% of the initial caffeine within the same period. Attending to these results a high percentage of initial caffeine in the microcapsules (about 78–80%) remained unaltered at 25 °C after 6 days of delivery suggesting a strong caffeine–MOF affinity. For the current application, this is considered a good result, since the goal is to achieve a perdurable stay of caffeine in the MOF and then in the polyamide yarn. This quantitative determination of caffeine in solution allowed the confirmation of the caffeine loading, inferring the viability of its release in a controlled way.

### 3.5 Study of the Caffeine Content in Composite Polyamide-6 Textile Fibers

Table 3 shows the percentage of capsules (zeolite Y, ZIF-8 and MIL-53(Al)) that the polyamide fibers could admit without altering the standard spinning process in Nurel S.A. The initial values of caffeine in the capsules and those analyzed in the composite fibers are also provided.

**Table 3:** Caffeine Analyzed by GC/MS in Fabrics of Fibers Containing Zeolite Y, ZIF-8, and MIL-53(Al) Nitrate as Capsules

material	caffeine at scaled-up capsules (wt %)^a^	capsules in the fiber (wt %)	theoretical caffeine, initial values (ppm)	caffeine analyzed in the fibers (ppm)^b^	remaining caffeine (%)
Zeolite Y	14	0.28	392	265	67.6
ZIF-8	26	0.60	1560	600	38.5
MIL-53(Al) nitrate	35	0.35	1225	1119	91.4
		0.45	1575	1489	94.5
		0.70	2450	1722	70.3

a Tested by TGA.

b Tested by GC/MS.

After the spinning process, a percentage of the additive hosted inside the porous materials was removed due to the extreme spinning conditions (260 °C). As shown in Table 3, attending to zeolite Y, 68% of the theoretical caffeine (caffeine at scaled-up capsules × capsules in the fiber) is analyzed in the fiber, while only 39% is envisaged for ZIF-8. Concerning MIL-53, from 0.35 to 0.70 wt % of CAF@MIL-53 capsules was incorporated in PA-6 fibers, resulting in a remaining caffeine in the garment of 70% for the higher percentage of capsules in the fiber. However, when the capsules were reduced to 0.35–0.45 wt % the remaining caffeine rose to 91–95%, corresponding to about 1100–1500 ppm of caffeine detection.

Table 4 shows the caffeine (ppm) detected after washing, scouring, and staining the PA-6 fabric containing the CAF@MIL-53(Al) capsules. The composite fabrics were first treated under washing machine conditions to analyze the behavior of the caffeine after this process (temperature of 30 °C, neutral soap, and 90 min). Up to 81 ppm of caffeine was detected when 0.70 wt % of CAF@MIL-53(Al) was in the fiber. This corresponds to 4.7% of the caffeine that remained after washing. It can be noted that a similar percentage (4.5%, corresponding to 51 ppm) was obtained when the content of capsules was reduced to 0.35 wt %. Compared with the release study of CAF@MIL-53(Al) capsules, it can be envisaged that an important percentage of the additive hosted inside the MIL-53(Al) formulating the polymeric fibers was removed due to the extreme spinning conditions (260 °C). Moreover, the effect of caffeine water solubility has a high impact. As seen in Table 4, it was found that in the CAF@MIL-53(Al) fabric with 0.45 wt % of MIL-53(Al), when scouring takes place only 6.9% of the initial caffeine remains. In any event, it can be stated that there is a certain amount of caffeine that is retained in the fibers and its dosage is slow; when the fibers with MIL-53(Al) are stained in blue, 1.3% and 1.7% of the initial caffeine remain for the fabrics with 0.35 and 0.70 wt % of capsules, respectively. The remarkable conclusion is that even if most of the additive was sacrificed along the different severe treatments, a detectable amount of caffeine endured due to its interaction with MOF microporous structure. In any case, tests under realistic conditions of use of the fabrics would be needed to study the cosmetic benefit of the additive.

**Table 4:** Caffeine Analyzed by GC/MS in Fabrics of Fibers Containing Capsules of MIL-53(Al) Nitrate after Washing, Scouring, and Staining

		caffeine in the composite PA-6 fibers after:	
material	capsules in the fiber (wt %)	washing^a^ (ppm)	scouring^b^/staining^c^ (ppm)
MIL-53(Al) nitrate	0.35	51	15^c^
	0.45		103^b^
	0.70	81	30^c^

a Washing conditions (neutral soap and water at 30 °C for 90 min).

b Scouring conditions (liquid detergent and water at 40 °C for at least 10 min).

c Souring + staining (Turquoise Dye in deionized water at 100 °C for 60 min).

### 3.6 Characterization of CAF@MIL-53(Al) Polyamide-6 Fibers

CAF@MIL-53(Al) fibers were characterized in the textile laboratory following the usual quality control procedure. Good mechanical properties and good behavior in the spinning process were found by using the masterbatch of 0.45 wt % of CAF@MIL-53(Al). These new functionalized fibers showed tenacity in the range of 3.1–3.3 versus 3.6 cN/dtex or standard fibers, and an elongation break of 64–71% (being 70% for conventional nylon fibers). When adding 0.70 wt % of capsules, the mechanical properties were also acceptable. A tenacity of 2.6 cN/dtex and an elongation break of 56% was measured. It can be noted, as advised in Table 3, that by increasing the percentage of capsules in the fiber, the caffeine tested on it was slightly reduced, probably because the capacity of assimilation of capsules in the fabric diminishes.

Morphological characterization of the fibers was also carried out. Figure S9 depicts a picture of the composite PA-6 fiber fabrics containing 0.35 and 0.70 wt % CAF@MIL-53 capsules (as prepared), 0.45 wt % CAF@MIL-53 capsules after scouring and 0.70 wt % CAF@MIL-53 capsules after blue staining. Fibers containing the highest amount of caffeine, 0.70 wt % capsule content (i.e., 1722 ppm) were further observed by SEM. Figure S10 shows the fibers woven into the garment once they were freeze fractured with liquid nitrogen: longitudinal view (Figure S10a), with fiber diameters of about 15–20 μm, and cross-section (Figure S10b). Figure S10c shows an individual MIL-53(Al) particle of about 500 nm embedded in the PA-6 matrix. The difficulty to observe the MOF relies on two reasons: (i) the low concentration of MOF and (ii) the perfect integration of the MOF (due to its chemical affinity) into the PA-6 fibers not showing MOF-polymer borders.

**Figure 7:**
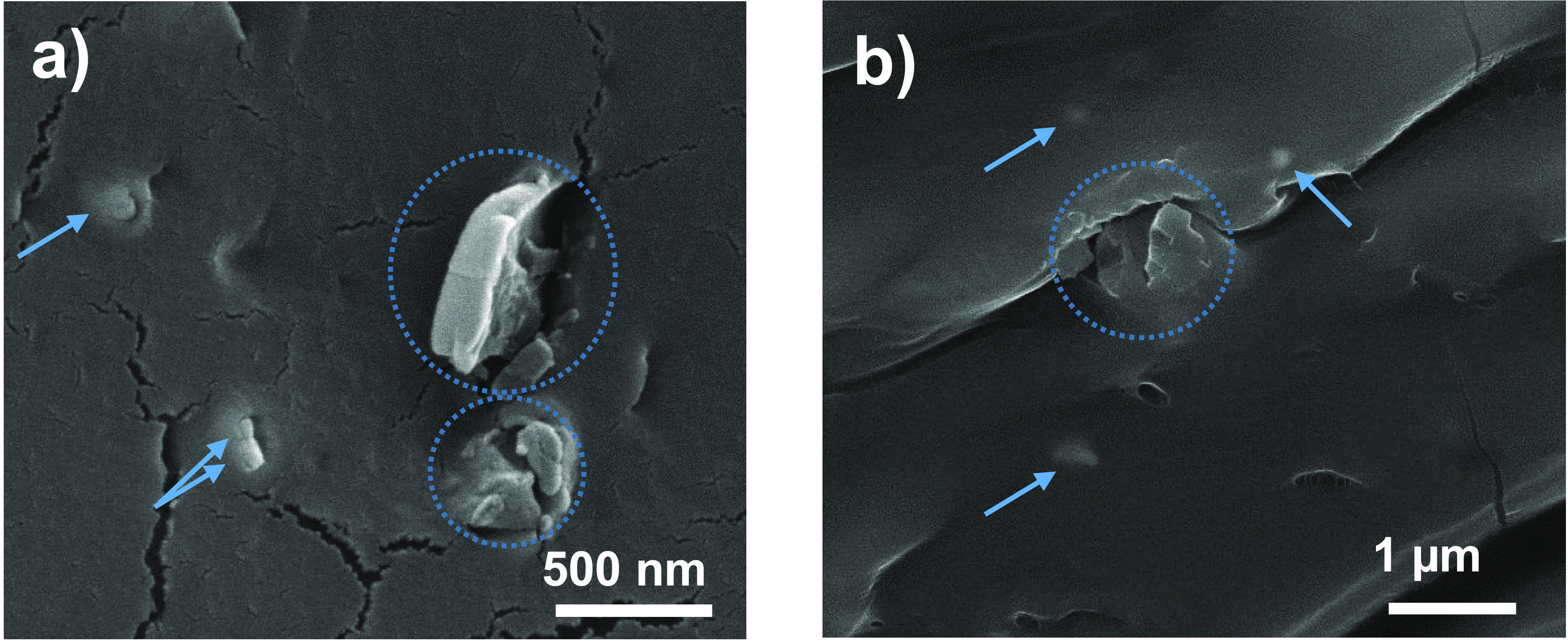
(a,b) SEM images of two different sections corresponding to 0.70 wt % CAF@MIL-53 PA-6 fibers.

To deepen into the visualization of the MIL-53 (Al) particles, the fabric was embedded in an epoxy resin and cut in slices. Figure 7 shows two different sections corresponding to the 0.7 wt % CAF@MIL-53 PA-6 fibers. MIL-53 particles distributed along the fibers are highlighted with circles. The arrows in the figure reveal smaller particles (about 200 nm) corresponding to TiO_2_, which was used as a white pigment added during the spinning process. The elemental composition of the particles was additionally corroborated by EDX analysis (not shown). The mapping for Al (coming from the MIL-53(Al) MOF) and Ti (from TiO_2_ nanoparticles) of Figure 7a,b is drawn in Figure S11a,b, respectively, within the selected region.

**Figure 8:**
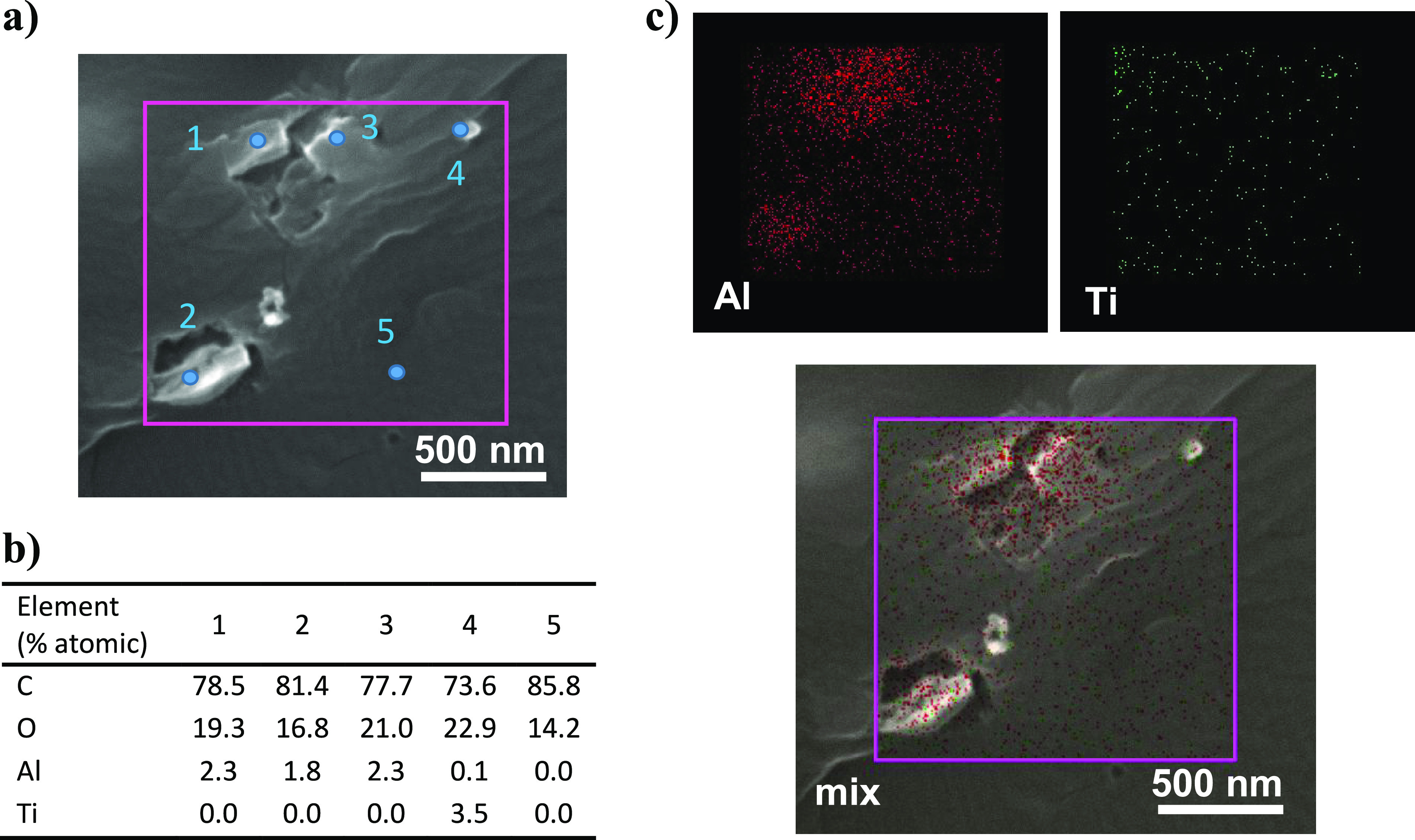
SEM-EDX study of a PA-6 fiber section containing 0.70 wt % CAF@MIL-53. (a) SEM image. (b) Elemental analysis of five points. (c) EDX mapping of the selected area. Al from MIL-53(Al) is depicted in red and Ti from TiO_2_ particles in green.

Finally, Figure 8a shows a SEM image of another fiber section containing several particles of MIL-53(Al) dispersed within the PA-6 polymer and highlighted as points 1, 2, and 3. Point 4 corresponds to a TiO_2_ particle, while 5 is pointing the PA-6 matrix. Figure 8b shows the EDX analyses for the five selected areas. It has to be taken into account that the proximity of points could alter their elemental composition, or even there might be some hidden particle underneath considering that the slides are about 1.5 μm thick. Approximately 2% of Al was detected in points 1–3 while points 4 and 5 did not reveal Al. Note that N was not taken into account in the atomic percentage, present in the caffeine, the PA-6, and the epoxy resin. Figure 8c shows the corresponding mappings for Al and Ti demonstrating the color mixing in the final SEM image.

## 4 Conclusions and Final Remarks

In this work, CAF@MIL-53(Al) capsules have been developed and applied to produce functionalized textile polyamide fibers. MIL-53(Al) was synthesized at different scaling-up levels (from the lab, about 1 g, to lab demonstrator, about 0.1 kg, production) exploring several MOF activation protocols to reduce the presence of unreacted linker molecules within the pores (BDC = 1,4-benzene dicarboxylate). Green solvents, such as water or the water/methanol mixture, have been used for the synthesis, producing the latter one practically activated MIL-53(Al) particles, which avoids the need for thermal or chemical activation treatments. In addition, two different Al sources were used in the MOF synthesis, Al(NO_3_)_3_·9H_2_O and Al_2_(SO_4_)_3_. Using sulfate salt instead of nitrate salt with the same aluminum molar ratio allowed: (i) reducing material costs, (ii) diminishing the pressure achieved in the reactor during the MOF synthesis, and (iii) producing more environmental friendly microcapsules. However, only the MIL-53(Al) prepared with nitrate salt was profitable to scaled-up. For the qualitative and quantitative determination of caffeine, several techniques (TGA, FTIR, XRD, GC-MS, UV–vis) were used. CAF@MIL-53(Al) particles and fabrics knitted with yarns of CAF@MIL-53(Al) microcapsules state a significant presence of the caffeine additive (up to 38 wt % in the microcapsules) incorporating a high amount of microcapsules in the polyamide spinning process (up to 0.70 wt %, measuring by GC-MS up to 1722 ppm of caffeine in the fabric). Only 22% of the initial caffeine, tested by UV–vis, was released from the CAF@MIL-53(Al) microcapsules at room temperature in an aqueous medium for 6 days while achieving a complete caffeine extraction at 80 °C for 12 h). As seen by SEM, CAF@MIL-53(Al) particles were evidenced in the fabric. EDX and mapping analyses corroborated the presence of Al from the MOF particles. The influence of washing, scouring, and staining stages on the additive permanence in the corresponding composite PA-6 fibers was studied, detecting an appreciable amount of caffeine in all the cases. Once the most superficial caffeine is released, the fraction embedded in the MIL-53 capsules practically endures over time, additionally remaining protected inside the fibers. This result is of relevant importance for their application in cosmetics in the progress toward the industrialization of new materials.

This work combines the experiences in the synthesis and characterization of zeolites and related materials, specifically MOFs, at lab-scale in a university research group with the experiences of two companies: NUREL, S.A., in the manufacture and commercialization of textile polyamide, and IQE, S.A., in the large-scale manufacture of silicates and zeolites. This tripartite collaboration is a good example of interaction between academy and industry. The textile fibers formed here gave rise to an enhancement in the process and the effectiveness of the functional fibers of NUREL together with the aperture of IQE toward new products and markets. The use of MOFs as alternative materials to zeolites provided greater encapsulation capacity, while a selection of MIL-53(Al) based on carboxylate MOF was validated at the pilot plant scale. From here, the research work continues in the search for new structures that allow encapsulation of different cosmetic additives to obtain a wide catalog of products able to manufacture.

## Supporting Information Available

Scheme of the industrial spinning process; TGA and XRD analyses of MIL-53(Al) and CAF@MIL-53(Al) samples; SEM images of MIL-53(Al) particles; TGA analyses of caffeine encapsulation in zeolite Y and ZIF-8; Photograph of CAF@MIL-53 PA-6 fabrics; SEM images and EDX mapping of CAF@MIL-53 PA-6 fibers (PDF)
