# Supplementary material for: Caffeine Encapsulation in Metal Organic Framework MIL-53(Al) at Pilot Plant Scale for Preparation of Polyamide Textile Fibers with Cosmetic Properties
Source: ACS Appl Mater Interfaces. 2022 May 4;14(19):22476–88. doi: 10.1021/acsami.2c04293 (PMC9121351; doi:10.1021/acsami.2c04293)
Supplement: Supplementary file 1 — am2c04293_si_001.pdf [file am2c04293_si_001.pdf]

# Caffeine encapsulation in metal organic framework MIL-53(Al) at pilot plant scale for preparation of polyamide textile fibers with cosmetic properties

## Supporting Information

*Beatriz Zornoza*[*,†,‡], *César Rubio*[†,‡], *Elena Piera*[§], *Miguel A. Caballero*[§], *Daniel Julve*[#], *Jorge Pérez*[#], *Carlos Téllez*[*,†,‡], *Joaquín Coronas*[†,‡].

[†] Instituto de Nanociencia y Materiales de Aragón (INMA), Universidad de Zaragoza-CSIC, 50009, Zaragoza, Spain

[†] Chemical and Environmental Engineering Department, Universidad de Zaragoza, 50018, Zaragoza, Spain

[§] Research and Development Department. Nurel S.A., Ctra. Barcelona km 329. 50016, Zaragoza, Spain

[#] Industrias Químicas del Ebro (IQE) S. A. Grupo IQE. 50016, Zaragoza, Spain

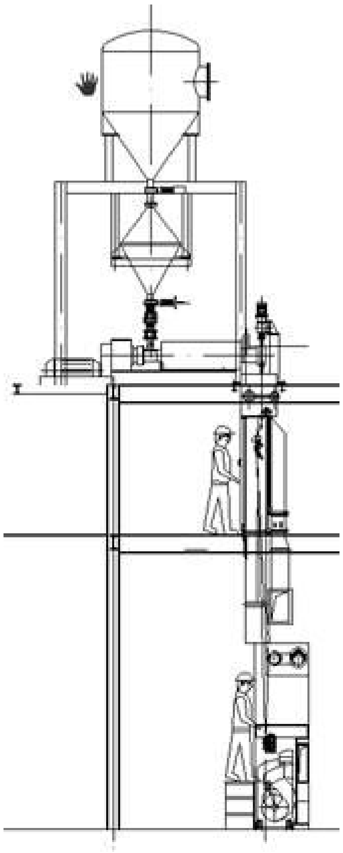

**Figure S1.** Scheme of the industrial spinning process for the preparation of composite polyamide fibers with cosmetic properties.

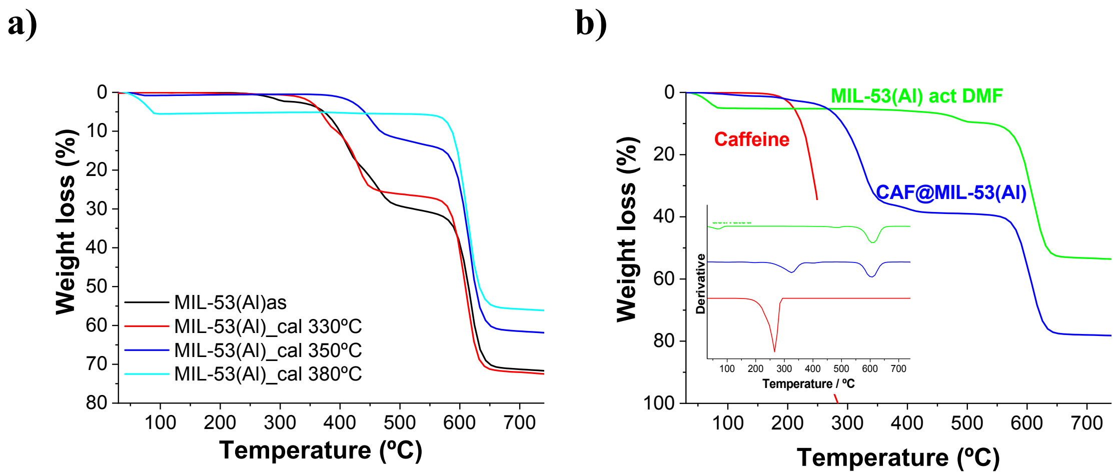


**Figure S2.** TGA analysis of the MIL-53(Al) samples synthesized in water at 220 ºC for 72 h: a) activation by calcination at different temperatures, and b) activation by DMF treatment and CAF@MIL-53(Al) compared with caffeine.

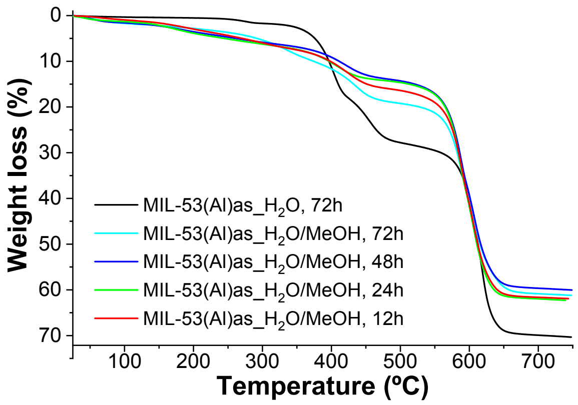


**Figure S3.** TGA analysis of MIL-53(Al) synthesized in water/methanol mixture at 150 ºC: a study of different reaction times from 12 to 72 h. MIL-53(Al)as, synthesized in water at 220 ºC for 72 h is included for comparison.

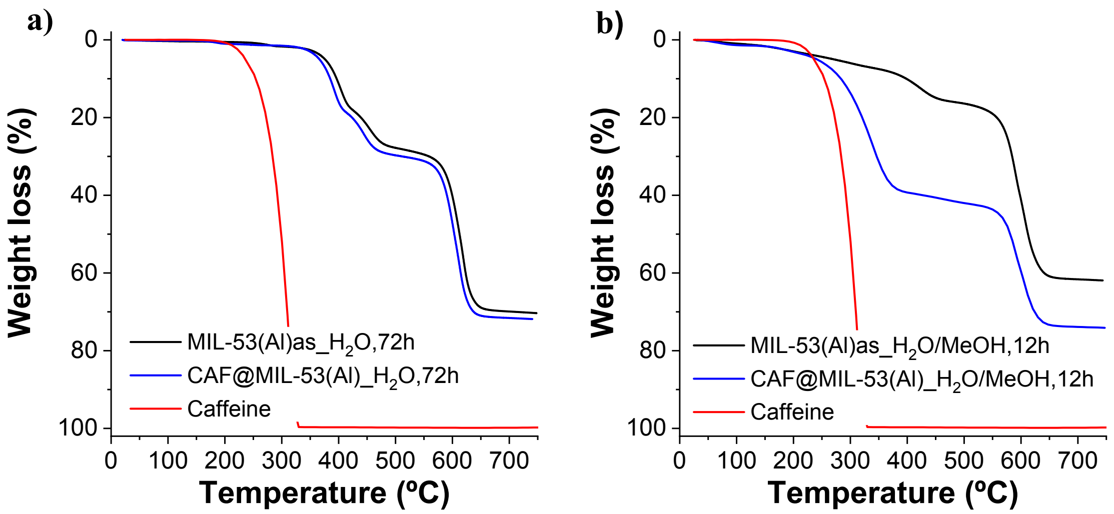


**Figure S4.** TGA analysis of MIL-53(Al) and CAF@MIL-53(Al) materials prepared without activation: a) synthesis in water (at 220 ºC for 72h, where activation is required for proper encapsulation), and b) synthesis in a water/methanol mixture (at 150 ºC for 12h, where activation is not needed and encapsulation takes place). Caffeine is included in both for a proper comparison.

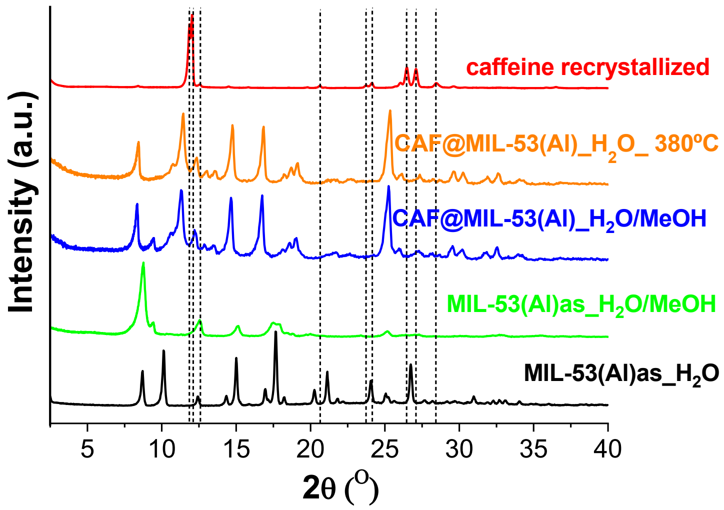


**Figure S5.** XRD of the different MIL-53(Al) synthesis: as-synthesized in water, as-synthesized in water/methanol mixture; caffeine encapsulation after calcination of MIL-53(Al) synthesized in water and caffeine encapsulation on MIL-53(Al) prepared in water/methanol mixture, without further activation.

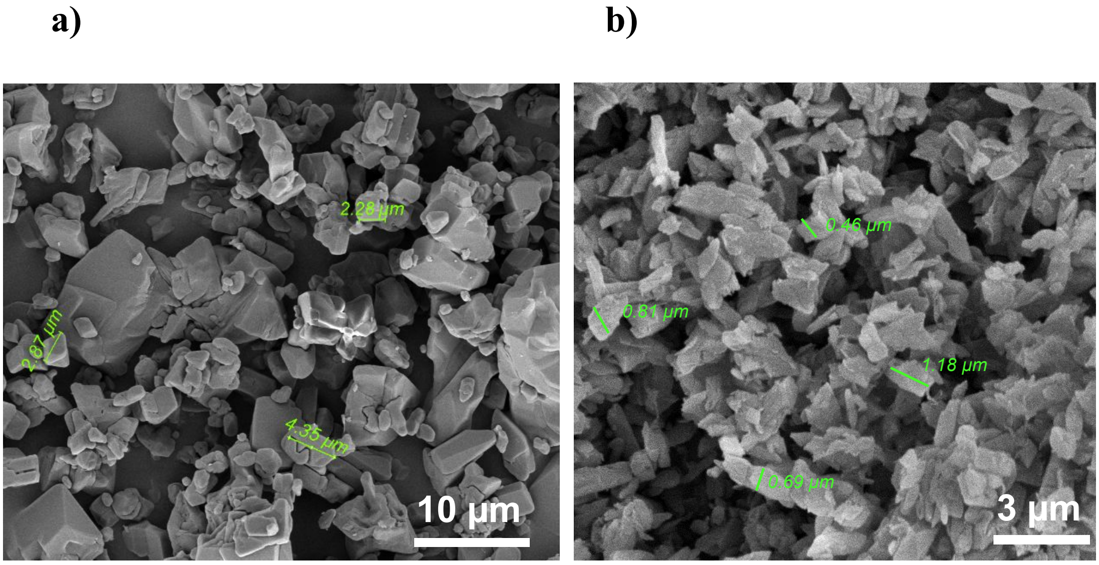


**Figure S6.** SEM images of MIL-53(Al) particles: a) synthesized in water as solvent, and b) synthesized by using water/methanol mixture.

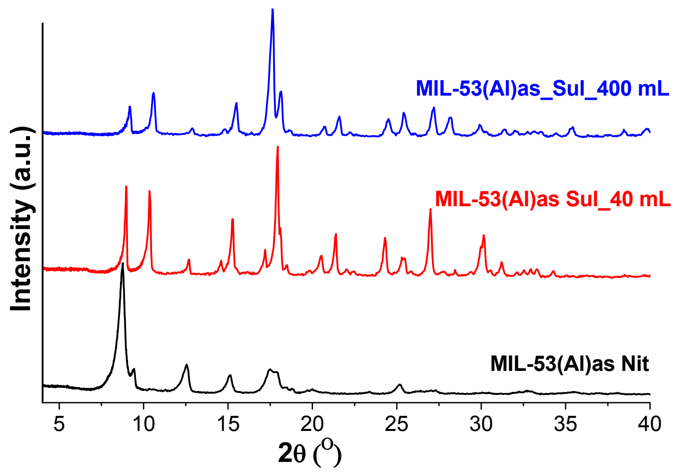


**Figure S7.** XRD patterns of the synthesis prepared at the reactor of 40 and 400 mL with SUFAL®8.2 compared with the synthesis of MIL-53(Al) prepared with aluminum nitrate using water/methanol mixture as solvent.

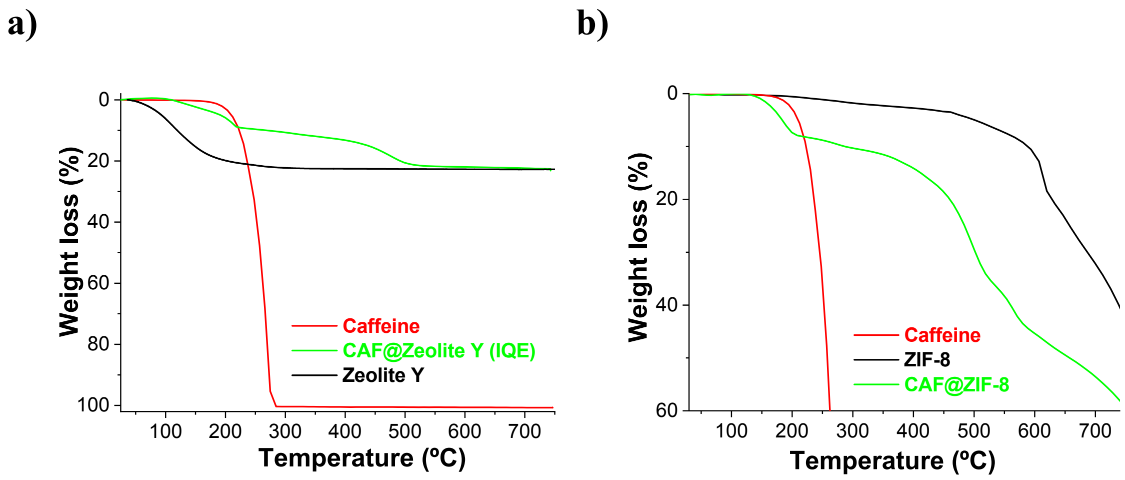


**Figure S8.** TGA analysis of caffeine encapsulation in a) zeolite Y, and b) ZIF-8. Note that zeolite Y needs to be thermally treated before additive encapsulation to remove adsorbed water.

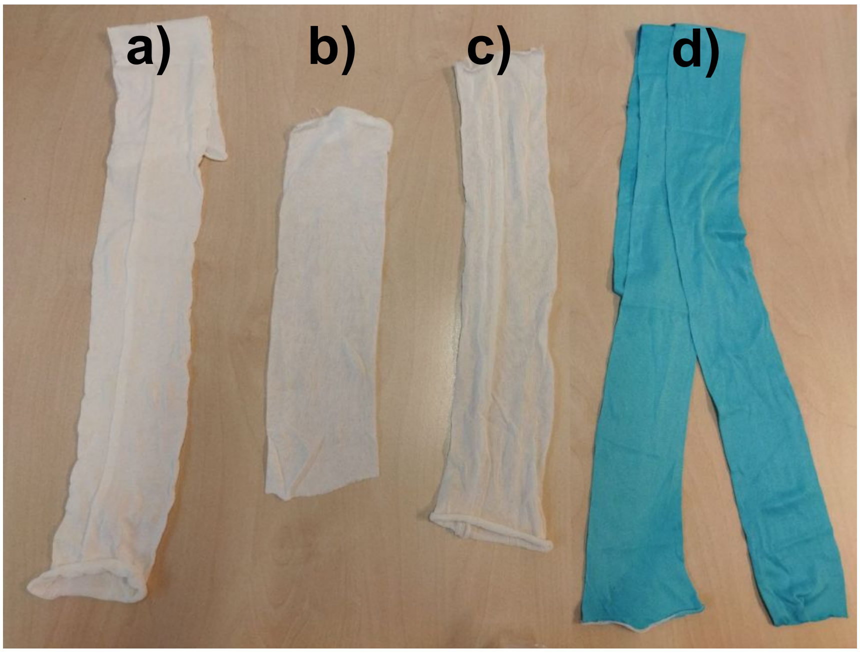


**Figure S9.** Photograph of CAF@MIL-53 PA-6 fabrics containing: a) 0.70 wt.% capsules, as prepared, b) 0.35 wt.% capsules, as prepared, c) 0.45 wt.% capsules, scoured, and d) 0.70 wt.% capsules, blue stained.

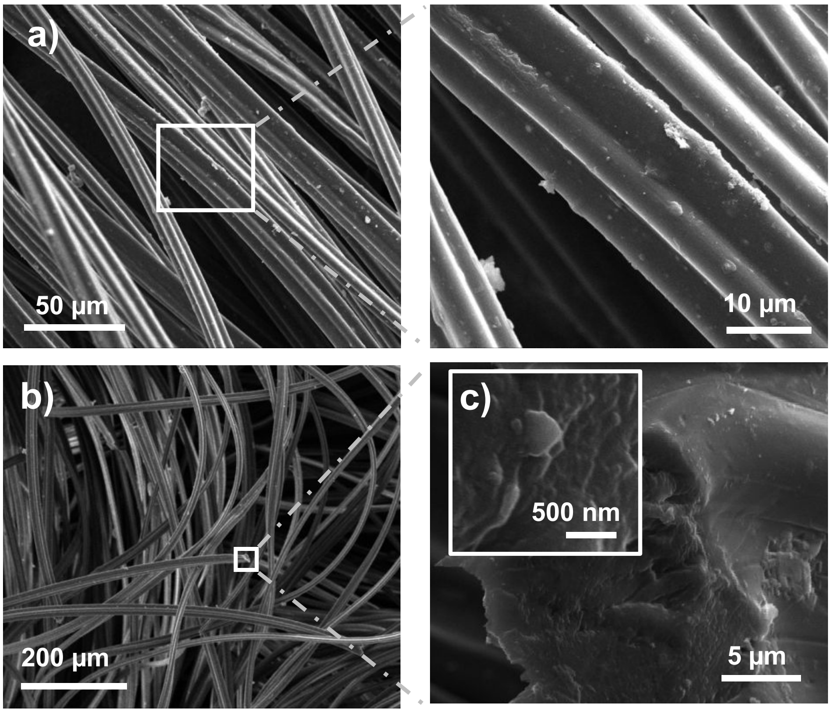


**Figure S10.** SEM images of 0.70 wt.% CAF@MIL-53 PA-6 fibers: a) longitudinal view and inset (right), and b) cross-section and inset (right); c) MIL-53(Al) individual particle embedded in the PA-6 fiber.

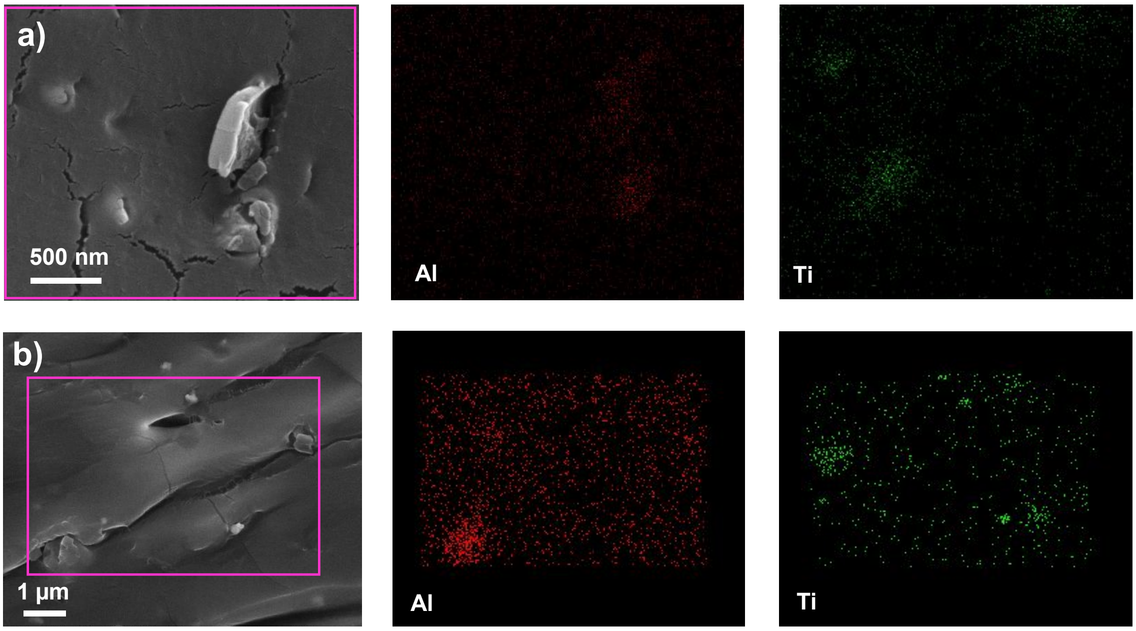


**Figure S11.** SEM image of two composite PA-6 fiber sections (a and b) containing 0.70 wt.% MIL-53(Al) and their corresponding EDX mapping. Al from MIL-53(Al) is visualized in red and Ti from $TiO_2$ particles is shown in green.
